# Validation of the Multidimensional Assessment of Interoceptive Awareness (MAIA-2) questionnaire in hospitalized patients with major depressive disorder

**DOI:** 10.1371/journal.pone.0253913

**Published:** 2021-06-25

**Authors:** Michael Eggart, Jennifer Todd, Juan Valdés-Stauber

**Affiliations:** 1 Department of Psychiatry and Psychotherapy I, Ulm University and Center for Psychiatry Südwürttemberg, Ravensburg, Germany; 2 Faculty Social Work, Health and Nursing, Ravensburg-Weingarten University of Applied Sciences, Weingarten, Germany; 3 School of Psychology and Sport Science, Anglia Ruskin University, Cambridge, United Kingdom; 4 Centre for Psychological Medicine, Perdana University, Kuala Lumpur, Malaysia; University of Bologna, ITALY

## Abstract

**Objectives:**

Interoception refers to the sensation, interpretation, and integration of internal somatic signals. Abnormalities in self-reported interoception are prevalent features of major depressive disorder (MDD) and may affect treatment outcomes. In the present study, we investigated the psychometric properties of the revised eight-dimensional and 37-item Multidimensional Assessment of Interoceptive Awareness questionnaire (the MAIA-2) in a severely depressed sample, after translating two updated scales (Not-Distracting, Not-Worrying) into German. Specifically, we examined the measure’s internal consistency reliability, sensitivity to change, and minimal important differences (MID) with a focus on patient’s antidepressive responses to treatment.

**Methods:**

The study enrolled 110 participants (age: *M* = 46.85, *SD* = 11.23; female: 55.45%) undergoing hospital treatment, of whom 87 were included in the pre-post analysis. Participants completed a German translation of MAIA-2 and the Beck Depression Inventory-II (pre-/post-treatment). Internal consistency reliability was determined by Cronbach’s *α*/McDonalds’s *ω*, sensitivity to change was determined by effect sizes, and MIDs were determined by distribution- (0.5*SD) and anchor-based approaches (mean change method; ROC curve cut-points).

**Results:**

Depression severity reduced over the course of treatment (Median = -65.22%), and 34.48% of patients achieved remission. Reliability was appropriate for post-treatment (range of *ω*: .70-.90), but questionable for two pre-treatment scales (Noticing: *ω* = .64; Not-Distracting: *ω* = .66). The eight dimensions of MAIA-2 were sensitive to change (standardized response mean: .32-.81; Cohen’s effect size: .30-.92). Distribution-based MIDs (.38-.61) and anchor-based mean change MIDs (remission vs. partial response: .00-.85; partial response vs. nonresponse: .08-.88) were established on the group level. For six scales, ROC cut-points (remission: .00–1.33; response: -.20–1.00) demonstrated accurate classification to treatment response groups on the individual level.

**Conclusions:**

This study demonstrated the applicability of the MAIA-2 questionnaire in MDD. The updated version may have led to reliability improvements regarding the revised scales, but subthreshold reliability was evident prior to treatment. The measure’s dimensions were sensitive to change. MIDs were established that corresponded with antidepressive treatment outcomes. Our findings are consistent with a growing area of research which considers somatic feelings as key contributors to mental health.

## 1. Introduction

Interoception has been defined as the sense of the physiological condition of the body [1,2] and refers to the sensation, interpretation, and integration of signals “originating from within the body, providing a moment-by-moment mapping of the body’s internal landscape across conscious and unconscious levels” [3] (p. 501). The primary function of interoception is to maintain homeostasis [4]. However, increasing evidence suggests that interoceptive feedback is also involved in shaping emotional experience, cognitions, self-awareness, time perception, emotion regulation, and other processes supporting mental health [1,2,5–7]. Although German physiologists described the involvement of somatic feelings in the psychopathology of mental disorders as early as the eighteenth century by establishing the term *Gemeingefühl* [8] (over a century before Sherrington coined the term interoception [9]), a PubMed search shows an exponential growth of publications on interoception since the turn of the millennium. This is possibly due to neuroscientific research and experimental methods (e.g., Schandry’s heartbeat tracking task [10]) which have stressed the relevance of interoceptive abnormalities for the pathogenesis and treatment of mental disorders [3].

Major depressive disorder (MDD) is one of the most common mental disorders, affecting over 300 million people worldwide [11]. MDD is a leading cause of disability and is associated with increased morbidity and mortality rates [11]. The treatment of MDD is particularly challenging because approximately one-third of patients report persistent residual symptoms of depression [12]. Residual symptoms are of high clinical importance because they predict unfavorable outcomes such as chronicity (i.e., absence of remission over two years), recurrence of depression, suicidality, and impaired daily functioning [12,13]. Therefore, the main objective of depression therapy has shifted from a focus on treatment response (defined as ≥ 50% reduction in depression symptom severity) to the achievement of full remission [13]. Recent research has identified interoceptive predictors of post-treatment residual symptoms of MDD, which could serve as prognostic markers for treatment response [14]. Specifically, changes in self-reported facets of interoception during inpatient treatment (e.g., worrying about unpleasant body sensations, low body confidence, interoception-driven emotion regulation difficulties) predicted antidepressive treatment outcome in hospitalized patients. These effects were independent of somatic symptom burden (e.g., pain) [14]. Cumulative evidence also suggests that other dimensions of interoception may be altered in MDD [15–17]. For example, if moderately depressed subjects are asked to silently count and report the number of their heartbeats during a heartbeat perception task, they regularly perform worse than healthy controls by underestimating the true number of their heartbeats (“interoceptive accuracy”) [15,16]. Further evidence suggests that depressed patients exhibit hypervigilant and anxiety-driven awareness of somato-vegetative states, high somatic symptom burdens without organic cause, and negative attitudes towards their bodies [18–22]. Moreover, neuroscientific evidence suggests abnormal activity and altered functional connectivity in brain regions that are both related to interoception (e.g., insula, anterior cingulate cortex, orbitofrontal cortex) and mood regulation [23–25]. To sum up, interoceptive abnormalities might be considered as a core characteristic of MDD [15,17,26]. These findings underscore the clinical relevance of responsive instruments that enable researchers to assess subjective interoceptive states and changes over time in individuals with a diagnosis of MDD.

In this paper, we refer to “self-reported interoception” as a facet of interoception comprising self-evaluations and attentional styles towards subjective interoceptive states which are accessible to consciousness and can be thus gauged via self-report measures [3]. The Multidimensional Assessment of Interoceptive Awareness (MAIA) is a questionnaire measure which assesses multifaceted aspects of self-reported interoception via eight scales (i.e., Noticing, Not-Distracting, Not-Worrying, Attention Regulation, Emotional Awareness, Self-Regulation, Body Listening, Trusting). The parent version includes 32 items [27] and has been translated into more than 20 languages. The MAIA’s ability to differentiate between maladaptive and beneficial attention styles towards the body is a major strength of the questionnaire [28]. The instrument conceptualizes self-reported interoception as a multidimensional construct, which is of great interest for clinical research. Indeed, other scales (e.g. Porges’ Body Perception Questionnaire [29]) have been criticized for exclusively focusing on anxiety-related rather than non-judgmental, or regulatory aspects of self-reported interoception [30]. The MAIA scales have been employed in healthy and clinical populations. Preliminary evidence points to severe body mistrust, impaired self-regulative skills, and abnormal bodily self-focus in patients suffering from MDD [26,31]. At present, three studies have used the MAIA questionnaire in depressed samples, and each study demonstrated nuanced patterns of findings between individual subscales treatment effects [14,32,33] highlighting the potential of the instrument as a promising outcome measure in clinical trials. However, the psychometric properties of the MAIA questionnaire have never been explicitly investigated in samples of individuals with a diagnosis of MDD. In contrast, a plethora of studies have investigated the construct and criterion validity of the MAIA in non-depressed samples (for a review, see [34]). Problematic internal consistency of both the Not-Distracting and Not-Worrying scales has been repeatedly reported (Cronbach’s *α* < .70), and some studies have also found low internal consistency for the Noticing scale [34]. To address the issue of low internal consistency, a revised version of the MAIA (the MAIA-2) was recently developed [35]. The MAIA-2 includes additional items in the Not-Distracting and Not-Worrying subscales, because the number of items in a scale is one factor that can affect internal consistency reliability estimates [35].

In the present pre-post study, we translated the new MAIA-2 items into German and investigated preliminary psychometric properties of the MAIA-2 questionnaire in a sample of inpatients with a main diagnosis of MDD undergoing treatment-as-usual. The naturalistic design allowed the inclusion of patients with somatic and psychiatric comorbidities unless patients met exclusion criteria which have been described in the companion paper [14]. First, we planned to report descriptive statistics and results of item analyses on all items that were assessed prior to treatment. We expected acceptable item-total correlations for all items. Second, we estimated internal consistencies of MAIA-2 scales for pre- and post-treatment measures by determining McDonald’s *ω* (i.e., an estimate of internal consistency reliability that is appropriate for situations where the assumption of an essentially *τ*-equivalent model is not met, which is usually the case for psychological scales [36]). We also estimated Cronbach’s α to facilitate comparisons of scale reliabilities with previous studies that used the MAIA/MAIA-2 in healthy adults and samples of patients suffering from MDD. Reliability estimates were calculated pre- and post-treatment to allow for the detection of potential effects of psychiatric symptom burden on internal consistency. There is preliminary evidence that careless response styles are common in psychiatric patients, which in turn may affect internal consistency estimates of Likert scales (see discussion below) [37–39]. Accordingly, we expected appropriate post-treatment reliabilities for all scales (ω ≥ .70) and hypothesized a qualitative trend towards lower estimates for the pre-treatment condition. We also expected improved reliabilities for both revised scales compared to a depressed sample using the MAIA. Third, we conducted an exploratory analysis of the scale’s pre-/post-treatment intercorrelations and compared the results with the original validation study of MAIA-2 [35]. Finally, we evaluated the responsiveness of the questionnaire by investigating its ability to detect changes over the course of hospital treatment (sensitivity to change) and expected significant improvements on all scales. The validity of inferential statistical change analysis has been criticized in clinical medicine, because small changes on outcome measures may be statistically significant (e.g. by increasing the sample size), but clinically negligible (i.e. without clinical significance) for patients [40,41]. Therefore, we followed best practice guidelines and further determined the minimal important difference (MID) of MAIA-2 scores by using distribution-based and anchor-based approaches to enhance the interpretability of the measure for future use in depressed populations [42]. According to Guyatt et al. [42] (p. 377), “the MID is the smallest difference in score in the domain of interest that patients perceive as important, either beneficial or harmful, and which would lead the clinician to consider a change in the patient’s management”. As we outlined previously, preliminary evidence suggests that changes on MAIA-2 dimensions independently predict response to antidepressant treatment and may consequently be of paramount prognostic importance [14]. However, interoceptive MID cutoffs have not been investigated to date. MIDs were determined by referring to a clinical anchor that represents widely accepted response types to treatment (remission, partial response, non-response–see methods for further definitions) [13]. We were particularly interested in the minimum magnitude of change that differentiates remitters from partial responders due to the high prognostic relevance of remission [13]. In a further analysis, we also determined the MIDs for partial responders versus non-responders, since partial response is associated with beneficial effects on quality of life, functional status, and overall well-being [43].

## 2. Methods

This single-armed naturalistic pre-post study was part of a larger project investigating the effects of treatment-as-usual on multidimensional self-reported interoception in hospitalized patients suffering from MDD and shares a data base with a previously published companion paper [14]. The study was approved by the ethics committee of Ulm University (reference: 13/17) and conducted in accordance with the Declaration of Helsinki. Written informed consent was obtained from all patients before enrolment.

### 2.1 Procedure and participants

Depressed individuals who were admitted to an inpatient ward specialized for the treatment of mood disorders (at the Department of Psychiatry and Psychotherapy I, Ulm University, Ravensburg, Germany) were consecutively recruited in the study. Eligible participants had to meet criteria for MDD (F32.x, F33.x) according to the International Classification of Diseases, 10^th^ edition (ICD-10) [44]. Diagnoses were assessed by trained experts (clinical psychologists and psychiatrists). The main reasons for exclusion were psychotic symptoms, substance dependence, or insufficient knowledge of the German language. Pre-assessments took place within 48 hours after admission (t_0_: pre-treatment), and post-assessments took place within the 48 hours before discharge from hospital (t_1_: post-treatment). 110 patients with complete data sets were included in the pre-treatment analysis. At post-treatment, complete pre-post data were available for *n* = 87 patients, who were included in the pre-post analysis. Thorough descriptions of the study design, a study flow diagram, a dropout analysis, sociodemographic/clinical characteristics of the longitudinal sample, and a comprehensive synopsis of the treatment-as-usual components (pharmacotherapy, individual and group psychotherapy, nursing interventions etc.) are provided in the companion paper [14].

### 2.2 Measures

#### 2.2.1 Multidimensional Assessment of Interoceptive Awareness, Version 2 (MAIA-2)

The MAIA-2 questionnaire used in this study is an updated version of the 32-item MAIA which was translated and validated in German by Bornemann et al. [45]. In the present study, we translated 3 new items from the Not-Distracting scale and 2 new items from the Not-Worrying scale, which were taken from the recent revision of the English MAIA-2 [35]. The items were forward/backward translated by the first author in collaboration with W. Mehling (Osher Center for Integrative Medicine, University of California, San Francisco, USA) and checked for face validity. The item wording and scale assignments are shown in Table 2. During data collection, items were presented in a fixed order.

The MAIA-2 assesses multidimensional aspects of self-reported interoception and includes 37 items scoring on a six-point Likert scale (0 never, 5 always). Subscale scores are calculated by taking the arithmetic mean of the items on each scale. Higher scores indicate beneficial self-reported interoception. In a recently published validation study, the authors suggested an eight dimensional model of the MAIA-2, which was supported in a general population sample [35]. However, several authors have failed to replicate the eight-factorial model of the previous version (MAIA) across cultural contexts (for a review, see [34]). To the best of our knowledge, further dimensional investigations of MAIA-2 are not available yet. The MAIA-2 includes the following dimensions [35]: (i) Noticing (“awareness of uncomfortable, comfortable, and neutral body sensations”); (ii) Not-Distracting (“tendency not to ignore or distract oneself from sensations of pain or discomfort”); (iii) Not-Worrying (“tendency not to worry or experience emotional distress with sensations of pain or discomfort”); (iv) Attention Regulation (“ability to sustain and control attention to body sensations”); (v) Emotional Awareness (“awareness of the connection between body sensations and emotional states”); (vi) Self-Regulation (“ability to regulate distress by attention to body sensations”); (vii) Body Listening (“active listening to the body for insight”); (viii) Trusting (“experience of one’s body as safe and trustworthy”). The German version of the questionnaire as well as scoring instructions are provided in the supplementary material (S1 Questionnaire).

#### 2.2.2 Beck Depression Inventory-II (BDI-II)

The BDI-II is a self-report measure assessing depression severity in clinically depressed individuals. The unidimensional instrument is sensitive to change and includes 21 items that are rated on a 4-point Likert scale. Previous research demonstrated adequate validity and excellent internal consistency and test-retest reliability of the measure [46]. Several studies determined MIDs for the BDI-II [47] and identified the percent change to baseline as the most appropriate measure of change (e.g., a reduction of 17.50% corresponded with ‘feeling better’ in randomized controlled trials) [48]. In the present study, internal consistency was excellent: *ω*_pre_ = .90 [95% bias-corrected and accelerated confidence interval (95% CI_BCa_) .85, .92]; *ω*_post_ = .94 [95% CI_BCa_ .91, .96].

### 2.3 Data analysis

All statistical analyses were performed in R 3.6.1 [49]. Precision of scale means, change scores, effect sizes (e.g., Pearson’s correlation coefficient *r*), and reliability coefficients were determined by following a non-parametric bootstrap procedure (95% CI_BCa_) with *R* = 10,000 replications by using the R *boot/BootES* packages [50,51]. An exploratory data inspection was performed prior to analysis and relevant outliers could not be identified. The occurrence of missing data was prevented by requesting study participants to complete the questionnaires in their entirety. Patients who were lost to post-assessments were excluded from the analysis.

#### 2.3.1 Item analysis

Descriptive statistics for MAIA-2 items were examined, including the arithmetic mean (*M*), standard deviation (*SD*), median (*MD*), as well as measures of shape (skewness/kurtosis). For each item-scale assignment of MAIA-2, item-total-correlations were computed after correcting for item overlap by using the alpha function of the R package *psych* [52]. Item-total-correlations ≥ .30 were defined as acceptable [53].

#### 2.3.2 Reliability analysis

Internal consistency coefficients of the MAIA-2 dimensions were determined by McDonald’s *ω* and Cronbach’s *α* for pre- and post-treatment scores, with .50 ≤ *α/ω* < .60 classified as poor, 60 ≤ *α/ω* < .70 as questionable, 70 ≤ *α/ω* < .80 as acceptable, 80 ≤ *α/ω* < .90 as good, and *α/ω* ≥ .90 as excellent [54]. For the present study, adequate reliability was a priori defined as *α/ω* ≥ .70 [55]. The range of Cronbach’s *α* was also estimated after dropping each item from the analysis.

#### 2.3.3 Sensitivity to change

We calculated simple change scores relative to baseline including 95% CI_BCa_ for each MAIA-2 scale (change = post—pre). There are multiple available methods to assess sensitivity to change with different statistical conceptualizations accounting for error in change scores [56]. As recommended by Norman et al. [56], we calculated the standardized response mean (*SRM*, Eq 1) and Cohen’s effect size (*CES*, Eq 2). The *CES* was interpreted following threshold values provided by Cohen [57]: small (.20), medium (.50), large (.80).


$$SRM=\frac{M_{post}-M_{pre}}{{SD}_{change}}$$ (1)

$$CES=\frac{M_{post}-M_{pre}}{{SD}_{baseline}}$$ (2)

#### 2.3.4 Minimal important difference (MID)

The MIDs were determined by referring to a distribution-based and anchor-based approach [42]. Regarding the distribution-based approach, we calculated the 0.5*SD of the baseline scores for each MAIA-2 scale [58]. As per the anchor-based approach, we decided to use a clinical anchor that reflects response types to antidepressant treatment. Therefore, we first computed the percent change of depression severity on BDI-II at post-treatment relative to baseline. Negative change represents a reduction in depression severity over the course of hospital treatment and was classified following commonly accepted cutoffs [13]: remission (BDI-II_change_ ≤ -80%), partial response (-80% < BDI-II_change_ ≤ -50%), or nonresponse (BDI-II_change_ > -50%). For binary classifications required in the receiver operating characteristic (ROC) curve analysis (see below), *response* was defined as BDI-II ≤ -50% and *remission* as BDI-II ≤ -80%. The usefulness of the clinical anchor was validated by computing bivariate correlations with percent depression change for each MAIA-2 dimension (criterion: *|r|* ≥ .30) [59]. The anchor-based MIDs were established by (a) the mean change and (b) the ROC curve method. Regarding the mean change method, the MIDs were calculated by subtracting (a1) the mean change score of nonresponders from the mean change score of partial responders, and by subtracting (a2) the mean change score of partial responders from the mean change score of remitters, respectively. Regarding the ROC curve method, we determined the MID cut-points for each MAIA-2 dimension which maximizes the sum of sensitivity and specificity by referring to Youden’s index (Eq 3).


Youden′sindex=max(sensitivity+specificity−1) (3)

In the present study, we were particularly interested in the minimal change for each MAIA-2 scale over the course of treatment that optimally classifies (b1) patients being in remission (= ‘positive test’) vs. no remission (= ‘negative test’), and (b2) treatment responders (= ‘positive test’) vs. nonresponders (= ‘negative test’), respectively. Therefore, separate ROC curve analyses were conducted by plotting the true positive rate (sensitivity; y-axis) against the false positive rate (1 –specificity; x-axis). In doing so, the area under the curve (AUC) represents the discriminatory ability of MAIA-2 scale change which should be different from .50. According to recommendations by Turner et al. [60], the entire sample was included in the ROC analysis to maximize precision of MID estimates. The validity of the cut-points was further assessed by determining the accuracy (proportion of correctly classified cases; see Table 7 notes for further clarification) and the more robust Cohen’s *κ* which may be conceptualized here as the agreement between binary clinical outcome (remission/response) and ROC classification predicted from the MID cut-points of MAIA-2 dimensions [48]. The ROC curve analysis was performed with the R packages *OptimalCutpoints* [61] and *cutpointr* [62].

## 3. Results

### 3.1 Participant characteristics

Sociodemographic and clinical characteristics of the pre-treatment sample are displayed in Table 1. During the study period, 23 participants dropped out, mainly due to unplanned discharge or uncompleted questionnaires at post-treatment. The average treatment duration was *M* = 8.59 (*SD* = 4.24) weeks.

**Table 1:** Characteristics of enrolled participants (pre-treatment, *n* = 110).

Characteristics	*M (SD)* or *n* (%)
Age (years)	46.85 (11.23)
Sex (female)	61 (55.45%)
Body mass index (kg/m^2^)	26.36 (5.43)
School education	
≤ 9 years	27 (24.55%)
10 years	45 (40.91%)
≥ 11 years	38 (34.54%)
Highest level of education	
no vocational training	12 (10.91%)
vocational training	78 (70.91%)
academic degree	20 (18.18%)
Employment status	
housekeeping or family care	7 (6.36%)
Unemployed	20 (18.18%)
Employed	72 (65.45%)
Retired	11 (10.00%)
Living alone (yes)	33 (30.00%)
Romantic relationship (yes)	64 (59.26%)
Main diagnosis (ICD-10)	
Single depressive episode (F32)	34 (30.91%)
Recurrent depressive disorder (F33)	76 (69.09%)
Severity of depression (ICD-10)	
Moderate (F3x.1)	13 (11.82%)
Severe without psychotic symptoms (F3x.2)	97 (88.18%)
Beck Depression Inventory-II sum score at admission	31.46 (10.41)
Somatic comorbidity (yes)	36 (32.73%)
Number of somatic diseases (self-report)	1.35 (1.65)
Number of psychotropic drugs at admission (self-report)	1.42 (1.28)
Number of past psychiatric inpatient stays (self-report)	1.48 (2.16)

*M* = arithmetic mean; *SD* = standard deviation; *n* = sample size; *%* = relative frequency.

Depression symptom severity significantly reduced over the course of treatment, *M* = -64.48% [95% CI_BCa_ -69.54, -58.40] (*SD* = 26.36), *MD* = -65.22% [95% CI_BCa_ -75.00, -60.00] (25^th^ percentile = -86.67%, 75^th^ percentile = -48.48%). At discharge from hospital, a total of *n* = 30 (34.48%) patients were in remission (BDI-II_post_: *M* = 2.57, *SD* = 2.16), *n* = 34 (39.08%) showed partial response to treatment (BDI-II_post_: *M* = 11.47, *SD* = 4.24), and *n* = 23 (26.44%) were classified as nonresponders (BDI-II_post_: *M* = 23.39, *SD* = 10.09).

### 3.2 Item analysis

Descriptive statistics of the 37 MAIA-2 items including item wording, corrected item-total correlations, and distribution indices are shown in Table 2. Item-total correlations for all items were in the expected direction and above an acceptable threshold (≥ .30). Concerning the shape of distribution, item 2 was highly skewed (skew ≤ -1.00), and items 4, 5, 7, 8, 10, 11, 16, 28, 33, 34 as well as all items on the Emotional Awareness scale were moderately skewed (−1.00 < skew < -0,50 or 0.50 > skew > 1.00).

**Table 2:** Item analysis and descriptive statistics of MAIA-2 (pre-treatment, *n* = 110).

Scale/Items of MAIA-2	*M*	*SD*	*MD*	*Skew*	*Kurt*	*ITC*
**Noticing** 1. Wenn ich angespannt bin, merke ich, wo in meinem Körper die Anspannung auftritt. *// When I am tense I notice where the tension is located in my body*.	3.01	1.45	3.00	-0.48	-0.84	0.62
2. Ich merke es, wenn ich mich in meinem Körper nicht wohlfühle. *// I notice when I am uncomfortable in my body*.	3.78	1.16	4.00	-1.21	1.03	0.50
3. Ich merke, wo in meinem Körper ich mich wohlfühle. *// I notice where in my body I am comfortable*.	2.25	1.49	2.00	0.16	-1.06	0.52
4. Ich bemerke Veränderungen in meiner Atmung, zum Beispiel, ob ich langsamer oder schneller atme. *// I notice changes in my breathing*, *such as whether it slows down or speeds up*.	2.95	1.66	4.00	-0.59	-0.98	0.49
**Not-Distracting** 5. Ich ignoriere körperliche Anspannung oder Unwohlsein, bis diese stärker werden. *// I ignore physical tension or discomfort until they become more severe*.	1.49	1.32	1.00	0.82	-0.01	0.46
6. Ich lenke mich von unangenehmen Empfindungen ab. *// I distract myself from sensations of discomfort*.	1.93	1.23	2.00	0.40	-0.33	0.33
7. Wenn ich Schmerz oder Unbehagen empfinde, versuche ich mich durchzubeißen. *// When I feel pain or discomfort*, *I try to power through it*.	1.50	1.40	1.00	0.75	-0.29	0.46
8. Ich versuche, Schmerzen zu ignorieren. ^a^ *// I try to ignore pain*. ^a^	1.98	1.38	2.00	0.59	-0.56	0.44
9. Ich verdränge unangenehme Gefühle, indem ich mich auf etwas anderes konzentriere.^a^ *// I push feelings of discomfort away by focusing on something*. ^a^	1.83	1.17	2.00	0.47	-0.38	0.66
10. Wenn ich unangenehme Körperempfindungen spüre, dann beschäftige ich mich mit etwas anderem, um sie nicht spüren zu müssen. ^a^ *// When I feel unpleasant body sensations*, *I occupy myself with something else so I don’t have to feel them*. ^a^	1.96	1.20	2.00	0.63	-0.37	0.70
**Not-Worrying** 11. Wenn ich körperliche Schmerzen habe, ärgere ich mich. *// When I feel physical pain*, *I become upset*.	1.88	1.66	1.00	0.52	-0.99	0.41
12. Wenn ich mich unwohl fühle, fange ich an mir Sorgen zu machen, dass irgendetwas nicht stimmt. *// I start to worry that something is wrong if I feel any discomfort*.	2.06	1.47	2.00	0.35	-0.83	0.73
13. Ich kann unangenehme Körperempfindungen spüren, ohne dass sie mich beunruhigen. *// I can notice an unpleasant body sensation without worrying about it*.	2.41	1.45	2.00	0.13	-0.94	0.59
14. Ich kann ruhig und unbesorgt bleiben, wenn ich mich unwohl fühle oder Schmerzen habe.^a^*// I can stay calm and not worry when I have feelings of discomfort or pain*. ^a^	1.73	1.26	2.00	0.16	-1.04	0.59
15. Wenn ich Unbehagen oder Schmerzen empfinde, geht es mir nicht aus dem Kopf. ^a^ *// When I am in discomfort or pain I can’t get it out of my mind*. ^a^	2.01	1.31	2.00	0.10	-1.05	0.52
**Attention Regulation** 16. Ich kann auf meine Atmung achten ohne von dem, was um mich herum geschieht, abgelenkt zu werden. *// I can pay attention to my breath without being distracted by things happening around me*.	1.85	1.27	2.00	0.53	-0.09	0.72
17. Ich kann meiner inneren Körperempfindungen gewahr bleiben, auch wenn um mich herum eine Menge los ist. *// I can maintain awareness of my inner bodily sensations even when there is a lot going on around me*.	1.92	1.31	2.00	0.29	-0.55	0.71
18. Ich kann auf meine Körperhaltung achten, während ich mich mit jemandem unterhalte. *// When I am in conversation with someone*, *I can pay attention to my posture*.	2.24	1.28	2.00	-0.03	-0.96	0.61
19. Wenn ich abgelenkt bin, kann ich mit meiner Aufmerksamkeit zu meinem Körper zurückkehren. *// I can return awareness to my body if I am distracted*.	1.84	1.17	2.00	0.18	-0.76	0.66
20. Ich kann meine Aufmerksamkeit vom Denken auf das Spüren meines Körpers zurücklenken. *// I can refocus my attention from thinking to sensing my body*.	1.90	1.25	2.00	0.10	-1.06	0.73
21. Ich kann den gesamten Körper auch dann weiter bewusst wahrnehmen, wenn ich in einem Teil Schmerz oder Unbehagen empfinde. *// I can maintain awareness of my whole body even when a part of me is in pain or discomfort*.	2.35	1.34	2.00	-0.01	-0.79	0.58
22. Ich kann meine Aufmerksamkeit bewusst auf meinen Körper als Ganzes richten. *// I am able to consciously focus on my body as a whole*.	2.12	1.37	2.00	0.17	-0.88	0.71
**Emotional Awareness** 23. Ich bemerke, wie mein Körper sich verändert, wenn ich wütend bin. *// I notice how my body changes when I am angry*.	3.05	1.46	3.00	-0.59	-0.57	0.66
24. Wenn etwas in meinem Leben nicht stimmt, kann ich das in meinem Körper spüren. *// When something is wrong in my life I can feel it in my body*.	3.14	1.52	4.00	-0.55	-0.78	0.53
25. Ich merke, dass mein Körper sich anders anfühlt, wenn ich etwas Friedliches und Entspannendes erlebe. *// I notice that my body feels different after a peaceful experience*.	3.50	1.31	4.00	-0.99	0.31	0.85
26. Ich merke, dass meine Atmung freier und leichter wird, wenn ich mich wohlfühle. *// I notice that my breathing becomes free and easy when I feel comfortable*.	3.46	1.43	4.00	-0.94	0.00	0.78
27. Ich merke, wie mein Körper sich verändert, wenn ich glücklich oder fröhlich bin. *// I notice how my body changes when I feel happy / joyful*.	3.55	1.36	4.00	-0.93	0.09	0.90
**Self-Regulation** 28. Wenn mir alles zu viel wird, kann ich einen Ort der Ruhe in mir finden. *// When I feel overwhelmed I can find a calm place inside*.	1.42	1.19	1.00	0.61	-0.41	0.55
29. Wenn ich meine Aufmerksamkeit auf meinen Körper richte, empfinde ich ein Gefühl innerer Ruhe. *// When I bring awareness to my body I feel a sense of calm*.	1.59	1.23	1.50	0.48	-0.34	0.59
30. Ich kann meinen Atem dazu benutzen, innere Spannungen abzubauen. *// I can use my breath to reduce tension*.	1.96	1.29	2.00	0.12	-1.06	0.70
31. Wenn ich in meine Gedanken verstrickt bin, kann ich meinen Geist beruhigen, indem ich auf Körper und Atem achte. *// When I am caught up in thoughts*, *I can calm my mind by focusing on my body/breathing*.	1.55	1.24	1.00	0.48	-0.70	0.75
**Body Listening** 32. Ich höre auf meinen Körper, was er über meine emotionale Verfassung sagt. *// I listen for information from my body about my emotional state*.	1.95	1.37	2.00	0.18	-1.05	0.57
33. Wenn ich aufgebracht bin, nehme ich mir Zeit herauszufinden, wie mein Körper sich anfühlt. *// When I am upset*, *I take time to explore how my body feels*.	1.15	1.02	1.00	0.55	-0.62	0.71
34. Ich höre auf meinen Körper, um zu erkennen, was zu tun ist. *// I listen to my body to inform me about what to do*.	1.46	1.29	1.00	0.50	-0.79	0.77
**Trusting** 35. Ich bin in meinem Körper zu Hause. *// I am at home in my body*.	2.18	1.45	2.00	0.32	-0.76	0.79
36. Ich empfinde meinen Körper als einen sicheren Ort. *// I feel my body is a safe place*.	1.92	1.36	2.00	0.32	-0.76	0.86
37. Ich vertraue meinen Körperempfindungen. *// I trust my body sensations*.	2.31	1.33	2.00	0.03	-0.75	0.70

^a^ new item of MAIA-2 compared to MAIA.

Number of each item is identical with the English version of MAIA-2 (possible range of ratings: 0–5); items 5, 6, 7, 8, 9, 10, 11, 12 and 15 were reverse scored prior to averaging likert scales (formula: 5—item score).

*M* = arithmetic mean; *SD* = standard deviation; *MD* = median; *ITC* = item-total-correlation corrected for item overlap; *Skew* = skew; *Kurt* = kurtosis.

### 3.3 Reliability analysis

Internal consistencies and descriptive statistics for each MAIA-2 dimension are summarized in Table 3. Regarding pre-treatment measures, McDonald’s *ω* ranged from .64 to .86. Both the Noticing (*ω* = .64 [95% CI_BCa_ .51, .75]) and Not-Distracting scale (*ω* = .66 [95% CI_BCa_ .52, .75]) showed subthreshold reliability (*ω* < .70). Regarding post-treatment measures, McDonald’s *ω* ranged from .70 to .90 and indicated appropriate reliability for all scales. There was a trend for better reliability classification for the post-treatment compared to the pre-treatment condition: from ‘questionable’ to ‘acceptable’ for the Noticing and Not-Distracting scales; from ‘acceptable’ to ‘good’ for the Self-Regulation and Body Listening scales; and from ‘good’ to ‘excellent’ for the Trusting scale.

**Table 3:** Reliabilities and descriptive statistics of the MAIA-2 scales, pre- (*n* = 110) and post-treatment (*n* = 87).

MAIA-2	Time	Internal consistency			Descriptive Statistics	Mehling et al. (2018) (MAIA-2)^a^		Fissler et al. (2016) (MAIA) ^b^	
		*α* [95% CI_BCa_]	Range of *α*_*drop*_	*ω* [95% CI_BCa_]	*M* (*SD*) [95% CI_BCa_]	*α*	*M* (*SD*)	*α*	*M* (*SD*)
Noticing	pre	.64 [.50, .75]	.52-.60	.64 [.51, .75]	3.00 (1.00) [2.81, 3.18]	.64	3.34 (.90)	.57	2.78 (.78)
	post	.73 [.55, .85]	.64-.69	.73 [.55, .84]	3.32 (.95) [3.10, 3.49]			.73	3.20 (.85)
Not-Distracting	pre	.67 [.55, .76]	.58-.68	.66 [.52, .75]	1.78 (.79) [1.64, 1.93]	.74	2.06 (.80)	.59	2.13 (.90)
	post	.72 [.55, .83]	.66-.72	.70 [.48, .82]	2.08 (.80) [1.90, 2.24]			.63	2.33 (.80)
Not-Worrying	pre	.71 [.59, .80]	.59-.72	.72 [.61, .80]	2.02 (.98) [1.83, 2.20]	.67	2.52 (.85)	.62	2.43 (1.07)
	post	.68 [.52, .79]	.53-.68	.70 [.55, .80]	2.50 (.88) [2.31, 2.68]			.59	2.83 (1.00)
Attention Regulation	pre	.85 [.80, .89]	.83-.85	.86 [.79, .89]	2.03 (.94) [1.86, 2.21]	.83	2.84 (.86)	.85	1.86 (.63)
	post	.88 [.82, .92]	.86-.87	.88 [.81, .92]	2.74 (.93) [2.54, 2.92]			.89	2.86 (.87)
Emotional Awareness	pre	.86 [.80, .91]	.79-.88	.86 [.80, .91]	3.34 (1.13) [3.11, 3.54]	.79	3.44 (.96)	.87	2.90 (1.24)
	post	.87 [.80, .93]	.81-.87	.87 [.81, .93]	3.69 (.84) [3.49, 3.84]			.91	3.46 (.92)
Self-Regulation	pre	.74 [.64, .81]	.63-.72	.74 [.63, .81]	1.63 (.93) [1.46, 1.80]	.79	2.78 (1.01)	.87	1.63 (1.08)
	post	.82 [.71, .89]	.71-.85	.84 [.75, .90]	2.51 (.99) [2.30, 2.71]			.89	2.85 (1.00)
Body Listening	pre	.75 [.65, .82]	.58-.76	.76 [.65, .82]	1.52 (1.01) [1.33, 1.71]	.80	2.20 (1.17)	.75	1.50 (.80)
	post	.83 [.71, .88]	.66-.86	.84 [.76, .89]	2.49 (1.04) [2.26, 2.70]			.85	2.57 (1.00)
Trusting	pre	.85 [.77, .90]	.72-.85	.85 [.77, .90]	2.14 (1.21) [1.91, 2.36]	.83	3.37 (1.11)	.84	2.17 (1.07)
	post	.89 [.80, .93]	.72-.90	.90 [.85, .94]	3.05 (1.19) [2.78, 3.28]			.87	3.03 (.97)

^a^ = validation study of MAIA-2 (English version, *n* = 1090 visitors of the Science Museum London).

^b^ = Moderately depressed sample (*n* = 38) pre/post a mindfulness training using the German MAIA.

95% BCa CI = 95% CI_BCa_ = 95% bias-corrected and accelerated bootstrapped confidence interval (*R* = 10,000 replications); *α* = Cronbach’s alpha; *α*_*drop*_ = Reliability (Cronbach’s alpha) after dropping an item from the scale; *ω* = McDonald’s omega; MAIA-2 = Multidimensional Assessment of Interoceptive Awareness, second version (higher scores indicate higher self-reported interoception resp. less distraction and worry; scoring 0–5; items are averaged across scales), Version 2; *M* = arithmetic mean; *SD* = standard deviation.

To facilitate comparisons with internal consistency reliability estimates from two previous studies, we also determined Cronbach’s *α*. Post-treatment internal consistency reliability estimates of the scales were largely comparable to Mehling et al.’s MAIA-2 validation study [35], except for the higher internal consistency estimates for the Noticing and Emotional Awareness scales in our sample. Comparisons of both revised scales with Fissler et al.’s MDD sample using the German MAIA [32] revealed a trend for improvements on both revised scales: Not-Distracting (from ‘poor’ to ‘questionable’ [pre-treatment]; from ‘questionable’ to ‘acceptable’ [post-treatment]), Not-Worrying (from ‘questionable’ to ‘acceptable’ [pre-treatment]; from ‘poor’ to ‘questionable’ [post-treatment]). Moreover, we evaluated each item’s impact on Cronbach’s alpha in our sample and could not identify any item that would significantly improve scale internal consistency reliability after its removal.

### 3.4 Scale-scale correlations

Zero-order correlations between MAIA-2 scales for pre- and post-treatment scores are shown in Table 4. Prior to treatment, scale-scale correlations ranged from *r* = -.40 (between Noticing and Not-Worrying) to *r* = .68 (between Noticing and Emotional Awareness). We screened for correlations significantly differing from Mehling et al.’s validation study [35] and identified the bivariate association between Noticing and Not-Worrying as the only deviation (Mehling et al. [35]: *r* = -.09). Regarding post-treatment scores, scale-scale correlations ranged from *r* = .04 (between Not-Distracting and Not-Worrying) to *r* = .69 (between Attention Regulation and Body Listening). Correlations were consistently positive and slightly increased in magnitude (|*r*|) compared to the English validation study [35]. A large positive correlation was found between the Trusting and the Not-Worrying scale (*r* = .60; Mehling et al. [35]: *r* = .16) as well as between Trusting and Body Listening (*r* = .63; Mehling et al. [35]: *r* = .29). No significant scale-scale correlations were found regarding the revised Not-Distracting scale (pre-/post-treatment).

**Table 4:** Zero-order correlations between MAIA-2 scales (below the diagonal: pre-treatment, *n* = 110; above the diagonal: post-treatment, *n* = 87).

MAIA-2	N	ND	NW	AR	EA	SR	BL	T
**Noticing (N)**	-	.12 [-.10, .40]	.13 [-.07, .32]	.43 [.24, .63]	.63 [.47, .78]	.35 [.06, .58]	.40 [.18, .57]	.31 [.08, .50]
**Not-Distracting (ND)**	.04 [-.17, .24]	-	.04 [-.17, .26]	.14 [-.15, .40]	.18 [-.06, .48]	.22 [-.03, .46]	.17 [-.13, .37]	.24 [-.01, .43]
**Not-Worrying (NW)**	-.40 [-.56, -.22]	-.08 [-.30, .13]	-	.37 [.16, .55]	.19 [-.01, .38]	.37 [.10, .56]	.29 [.05, .48]	.60 [.44, .72]
**Attention Regulation (AR)**	.24 [.03, .42]	-.08 [-.26, .10]	.12 [-.08, .33]	-	.68 [.55, .79]	.60 [.43, .73]	.69 [.52, .79]	.63 [.47, .73]
**Emotional Awareness (EA)**	.68 [.54, .78]	.00 [-.21, .21]	-.37 [-.53, -.18]	.37 [.17, .54]	-	.56 [.35, .71]	.58 [.44, .70]	.52 [.36, .64]
**Self-Regulation (SR)**	.36 [.19, .50]	-.11 [-.29, .07]	.05 [-.16, .24]	.50 [.32, .63]	.39 [.20, .54]	-	.61 [.37, .75]	.63 [.43, .75]
**Body Listening (BL)**	.41 [.21, .56]	.14 [-.06, .32]	-.26 [-.42, -.07]	.46 [.27, .60]	.44 [.27, .57]	.45 [.26, .60]	-	.63 [.43, .76]
**Trusting (T)**	.27 [.06, .44]	.02 [-.18, .23]	.11 [-.09, .31]	.47 [.30, .61]	.22 [.02, .40]	.38 [.18, .54]	.33 [.14, .50]	-

95% bias-corrected and accelerated bootstrapped confidence intervals are reported in brackets (*R* = 10,000 replications).

### 3.5 Sensitivity to change

The instrument’s ability to detect change over the course of treatment was studied for each MAIA-2 scale. Findings are shown in Table 5. First, we calculated pre-post change scores and found significant improvements on all dimensions (as demonstrated by the confidence intervals excluding *M*_change_ = .00). Second, we performed a sensitivity to change analysis by determining standardized coefficients of change. The *SRM* ranged from .32 (Emotional Awareness) to .81 (Trusting). Values for *CES* were found to be largely similar and ranged from .30 (Emotional Awareness) to .92 (Body Listening). According to Cohen’s effect size classification [57], small improvements were found on the Noticing, and Emotional Awareness scales. Medium positive changes could be demonstrated for the Not-Distracting, Not-Worrying, Attention Regulation, and Trusting scales. Large improvements occurred on the Self-Regulation, and Body Listening dimensions.

**Table 5:** Sensitivity to change and distribution-based minimal important differences of the MAIA-2 scales (*n* = 87).

MAIA-2	Baseline score	Change score^a^	Sensitivity to change		MID_distr_
	*M* (*SD*) [95% CI_BCa_]	*M* (*SD*) [95% CI_BCa_]	*SRM* [95% CI_BCa_]	*CES* [95% CI_BCa_]	0.50∙*SD*_*pre*_ [95% CI_BCa_]
Noticing	2.92 (.96) [2.71, 3.10]	.40 (1.03) [.20, .62]	.39 [.19, .57]	.42 [.20, .63]	.48 [.42, .56]
Not-Distracting	1.67 (.76) [1.52, 1.84]	.40 (.92) [.21, .59]	.44 [.18, .67]	.53 [.25, .81]	.38 [.33, .45]
Not-Worrying	2.03 (.92) [1.84, 2.22]	.47 (.86) [.30, .65]	.54 [.34, .75]	.51 [.29, .75]	.46 [.40, .55]
Attention Regulation	2.05 (.99) [1.85, 2.26]	.69 (1.02) [.47, .90]	.67 [.45, .89]	.69 [.46, .96]	.49 [.43, .57]
Emotional Awareness	3.35 (1.12) [3.10, 3.57]	.33 (1.04) [.12, .55]	.32 [.11, .53]	.30 [.10, .50]	.56 [.48, .66]
Self-Regulation	1.66 (.97) [1.46, 1.86]	.86 (1.13) [.62, 1.09]	.76 [.51, 1.00]	.88 [.59, 1.16]	.49 [.43, .55]
Body Listening	1.56 (1.01) [1.35, 1.77]	.93 (1.21) [.67, 1.18]	.76 [.51, 1.00]	.92 [.63, 1.21]	.50 [.45, .57]
Trusting	2.17 (1.22) [1.92, 2.43]	.88 (1.09) [.66, 1.12]	.81 [.58, 1.02]	.72 [.51, .97]	.61 [.54, .70]

^a^ Positive change scores represent improvements in self-reported interoception over the course of the treatment (equation: change = post–pre).

*M* = mean; *SD* = standard deviation; 95% CI_BCa_ = 95% bias-corrected and accelerated bootstrapped confidence interval (*R* = 10,000 replications); *SRM* = standardized response mean; *CES* = Cohen’s effect size; MID_distr_ = minimal important difference (distribution-based approach).

### 3.6 Minimal important difference (MID)

First, we determined MIDs separately for each MAIA-2 scale by referring to the distribution-based method (Table 5). The 0.5*SDs ranged between .38 (Not-Distracting) and .61 (Trusting). In a next step, we evaluated the usefulness of the clinical anchor (percent reduction in depression severity) by computing bivariate correlations with change scores for each MAIA-2 dimension. Correlations with the Noticing (*r* = -.20 [95% CI_BCa_ -.39, .00]) and Not-Distracting scales (*r* = -.09 [95% CI_BCa_ -.31, .14]) were below an acceptable threshold (*|r|* ≥ .30). Therefore, findings for these scales should be interpreted with caution. The other scales met the correlational requirements of the anchor-based method: Not-Worrying (*r* = -.31 [95% CI_BCa_ -.50, -.07]), Attention Regulation (*r* = -.32 [95% CI_BCa_ -.46, -.10]), Emotional Awareness (*r* = -.31 [95% CI_BCa_ -.47, -.10]), Self-Regulation (*r* = -.50 [95% CI_BCa_ -.63, -.31]), Body Listening (*r* = -.47 [95% CI_BCa_ -.62, -.31]), Trusting (*r* = -.37 [95% CI_BCa_ -.53, -.14]).

Table 6 shows the mean change on all MAIA-2 dimensions and corresponding MIDs stratified by response types to antidepressant treatment. From nonresponse to partial response to remission, mean changes demonstrated a growing tendency towards improvements on all scales except for Not-Distracting. As per the mean change method, MIDs ranged between .08 (Attention Regulation) and .88 (Self-Regulation) for nonresponders vs. partial responders and between .00 (Not-Disctracting) and .85 (Attention Regulation) for patients who achieved remission vs. partial responders.

**Table 6:** Mean change scores of MAIA-2 and anchor-based minimal important difference stratified by treatment response (*n* = 87).

	MAIA-2 change scores^a^ *M* [95% CI_BCa_]							
	Noticing	Not-Distracting	Not-Worrying	Attention Regulation	Emotional Awareness	Self-Regulation	Body Listening	Trusting
**Nonresponse**^**b**^ **(*n* = 23)**	.07 [-.25, .39]	.29 [-.04, .63]	.12 [-.10, .38]	.34 [.03, .68]	-.08 [-.52, .37]	-.01 [-.44, .36]	.25 [-.26, .65]	.36 [-.04, .80]
**Partial Response**^**c**^ **(*n* = 34)**	.50 [.20, .90]	.44 [.20, .68]	.27 [.00, .51]	.41 [.09, .72]	.25 [-.06, .62]	.87 [.52, 1.18]	.82 [.44, 1.15]	.78 [.50, 1.07]
**Remission**^**d**^ **(*n* = 30)**	.55 [.17, .98]	.44 [.00, .83]	.96 [.62, 1.28]	1.26 [.91, 1.62]	.73 [.45, 1.09]	1.51 [1.18, 1.86]	1.57 [1.19, 2.02]	1.38 [.99, 1.82]
**No remission (*n* = 57)**	.33 [.09, .60]	.38 [.18, .58]	.21 [.03, .39]	.38 [.15, .61]	.12 [-.15, .40]	.51 [.22, .78]	.59 [.28, .85]	.61 [.36, .85]
**MID**_**change**_**: partial response—nonresponse**	.44 [-.01, .94]	.15 [-.26, .56]	.15 [-.22, .48]	.08 [-40, .53]	.33 [-.25, .89]	.88 [.35, 1.40]	.58 [.04, 1.15]	.42 [-.09, .93]
**MID**_**change**_**: remission—partial response**	.05 [-.50, .58]	.00 [-.49, .46]	.69 [.28, 1.12]	.85 [.37, 1.33]	.48 [.01, .94]	.64 [.19, 1.13]	.74 [.24, 1.31]	.58 [.12, 1.11]

^a^ Positive change (i.e. higher scores) indicates improved self-reported interoception (formula for change scores: post—pre).

^b^ Reductions on BDI-II > -50% relative to baseline were classified as nonresponse.

^c^ Reductions -80% < BDI-II ≤ -50% relative to baseline were classified as partial response.

^d^ Reductions on BDI-II ≤ -80% relative to baseline were classified as remission.

95% CI_BCa_ = 95% bias-corrected and accelerated bootstrapped confidence interval (*R* = 10,000 replications); *M* = mean; MAIA-2 = Multidimensional Assessment of Interoceptive Awareness, Version 2; MID_change_ = minimal important difference (mean change method, anchor-based approach; change scores show mean differences between groups: a) partial response vs. nonresponse; b) remission vs. partial response).

We also performed ROC curve analyses to establish optimal cut-points based on MAIA-2 changes that were predicting remission or treatment response by maximizing Youden’s index. The main findings are reported in Table 7. The ROC-derived MIDs ranged between ≥ .00 (Emotional Awareness) and ≥ 1.33 (Body Listening, Trusting) for achieving remission and between ≥ -.20 (Emotional Awareness) and ≥ 1.00 (Self-Regulation) for treatment response, respectively. Except for the Noticing and Not-Distracting scales, the AUCs were significantly different from .50 implicating non-random classifiers. The agreement between dichotomized clinical outcome (remission/response) and ROC classification predicted from cut-points was reasonable (*κ* ≥ .20) except for the treatment response vs. nonresponse groups on the Noticing and Not-Distracting scales (*κ* < .20). The best agreement was found for Attention Regulation (*κ* = .44) regarding achievement of remission and for Self-Regulation (*κ* = .40) regarding response to treatment.

**Table 7:** Cut-points for MAIA-2 scales determined by ROC curve analyses (*n* = 87).

MAIA-2	remission (*n* = 30) vs. no remission (*n* = 57)^a^						response (*n* = 64) vs. nonresponse (*n* = 23)^b^					
	MID_cut_	Se [95% CI]	Sp [95% CI]	AUC [95% CI]	Ac	*κ*	MID_cut_	Se [95% CI]	Sp [95% CI]	AUC [95% CI]	Ac	*κ*
**Noticing**	≥ .75	.53 [.34, .72]	.74 [.60, .84]	.58 [.45, .72]	.67	.27	≥ .75	.42 [.30, .55]	.83 [.61, .95]	.62 [.49, .75]	.53	.17
**Not-Distracting**	≥ .67	.50 [.31, .69]	.72 [.58, .83]	.56 [.42, .70]	.64	.22	≥ .17	.67 [.54, .78]	.48 [.27, .69]	.56 [.42, .70]	.62	.13
**Not-Worrying**	≥ .60	.67 [.47, .83]	.72 [.58, .83]	.73 [.62, .85]	.70	.37	≥ .40	.56 [.43, .69]	.78 [.56, .93]	.67 [.55, .79]	.62	.26
**Attention Regulation**	≥ .71	.80 [.61, .92]	.68 [.55, .80]	.74 [.64, .85]	.72	.44	≥ .86	.53 [.40, .66]	.83 [.61, .95]	.65 [.52, .77]	.61	.26
**Emotional Awareness**	≥ .00	.87 [.69, .96]	.47 [.34, .61]	.68 [.57, .79]	.61	.28	≥ -.20	.86 [.75, .93]	.48 [.27, .69]	.64 [.50, .79]	.76	.35
**Self-Regulation**	≥ 1.00	.80 [.61, .92]	.60 [.46, .72]	.75 [.64, .85]	.67	.35	≥ 1.00	.67 [.54, .78]	.83 [.61, .95]	.80 [.69, .91]	.71	.40
**Body Listening**	≥ 1.33	.53 [.34, .72]	.81 [.68, .90]	.71 [.60, .82]	.71	.35	≥ .33	.83 [.71, .91]	.52 [.31, .73]	.73 [.61, .84]	.75	.35
**Trusting**	≥ 1.33	.53 [.34, .72]	.81 [.68, .90]	.70 [.58, .81]	.71	.35	≥ .33	.75 [.63, .85]	.56 [.34, .77]	.69 [.55, .82]	.70	.29

^a^ Reductions on BDI-II ≤ -80% relative to baseline were classified as remission or ‘positive test’ (no remission: BDI-II > -80%).

^b^ Reductions on BDI-II ≤ -50% relative to baseline were classified as response or ‘positive test’ (nonresponse: BDI-II > -50%).

95% CI = 95% confidence interval; MID_cut_ = minimal important difference (anchor-based approach), cut-points determined with ROC curve analysis (by maximizing Youden’s index); Se = sensitivity; Sp = specificity; AUC = area under the curve; Ac = Accuracy, i.e. proportion of correctly classified cases ($\frac{\mathrm{True}\;\mathrm{Positive}+\mathrm{True}\;\mathrm{Negative}}{n}$); *κ* = Cohen’s *κ* (agreement between dichotomized clinical outcome (remission/response) and ROC classification predicted from cut-points).

## 4. Discussion

In the present study, psychometric properties of the German translation of the MAIA-2 were examined in a severely depressed inpatient sample. Specifically, we investigated the measure’s internal consistency reliability, scale intercorrelations, sensitivity to change, and MIDs operationalized as changes on the measure’s dimensions that were predictive of treatment responses.

The MAIA questionnaire is a widely used instrument that was designed to assess clinically relevant dimensions of self-reported interoception. However, several psychometric issues have been reported across both clinical and healthy populations, as well as across cultural contexts (for a comprehensive review, see [34]). For example, several studies have not replicated the eight-factor model of the MAIA [34]. To the best of our knowledge, the instrument’s factorial validity has never been investigated in patients suffering from MDD. In the present study, we were not able to run factorial analyses on our data to provide reliable estimates of the measure’s dimensionality due to limitations in sample size. Therefore, main results of the present analysis should be interpreted cautiously until sufficiently powered validation studies are available that confirm the dimensional structure of MAIA-2. Nevertheless, there is an urgent need for an investigation of preliminary psychometric properties of MAIA-2 due to its increasing clinical use and due to the high clinical impact of interoceptive disturbances in MDD [14,15,26,31–33].

### 4.1 Internal consistency of MAIA-2

First, we investigated the measure’s internal consistency reliability. Previously, psychometric concerns have been raised regarding low internal consistency on some scales, which led the principal investigators to publish a revision of the questionnaire by adding further items to the Not-Distracting and Not-Worrying scales [35]. These limitations appear to be particularly pronounced in a sample with an acute clinical condition [32]. However, a large proportion of studies have reported Cronbach’s *α* [34] which may not be an appropriate measure of internal consistency due to its reliance on essentially *τ*-equivalent models [36]. Therefore, previously reported reliability estimates may be at risk for bias—which is why this study also referred to McDonald’s *ω*. As expected, post-treatment reliabilities were appropriate for all eight dimensions of MAIA-2 (*ω* ≥ .70). However, the pre-treatment condition revealed questionable internal consistency of the Noticing and Not-Distracting scales (*ω* < .70). We did not expect further reliability constraints of the Not-Distracting dimension since the scale was revised and showed appropriate reliability in the original validation study [35]. In contrast, the Noticing scale has been criticized for problematic reliability in some studies [34], but validations using the German translation consistently demonstrated acceptable internal consistency [45,63]. As hypothesized, there was a qualitative trend towards better reliability classifications for the post-treatment measures which also applies to the latter two dimensions. A comparable pattern has been reported in another longitudinal study recruiting depressed patients [32]. In terms of theoretical mechanisms, it is possible that there is an interplay between psychopathological symptomology and the properties of internal consistency reliability: it has been shown that reliability of a self-report Likert scale may be affected by “insufficient effort responding” (careless responding styles), for example by “straightlining” identical responses or giving random ratings [37], resulting in biased estimates of internal consistency [38]. Accordingly, recent research has shown that psychiatric symptoms are associated with less consistent response patterns on self-rated scales [39]. Consequently, we argue that the potential heterogeneity across pre- and post-treatment reliability estimates might be attributed to depression-related psychopathology (e.g. cognitive impairments, motivational deficits, fatigue effects) resulting in insufficient effort responding and possibly biasing internal consistency estimates particularly in the pre-treatment condition. Notwithstanding this, the analysis could not identify any item that would give rise to significantly improved reliability after its removal.

Moreover, we compared internal consistency reliability estimates for each MAIA-2 dimension with estimates from previous studies. The main findings suggest that scale’s post-treatment internal consistency estimates were similar to Mehling et al.’s [35] validation study except for the Noticing and Emotional Awareness scales yielding significantly higher Cronbach’s *α* in our study [35]. However, similar internal consistency reliability estimates of both scales were reported in clinical samples [32,64,65]. Such heterogeneity across studies might be explained by differences in participant characteristics across studies (e.g. mental health status, experience in mind-body training, and linguistic or cultural contexts) [35]. The comparison of potential reliability improvements across heterogenous study conditions may be at risk for bias. For this reason, potential internal consistency improvements of the revised MAIA-2 scales (i.e., Not-Distracting and Not-Worrying) were also evaluated by comparing our findings with post-treatment Cronbach’s *α* coefficients from a clinically depressed sample by Fissler et al. using the German MAIA [32]. To minimize differences between studies, only post-treatment measures were included in the exploratory comparison because Fissler et al. investigated a moderately depressed, ambulatory sample. Post-treatment internal consistency estimates of both revised scales improved regarding MAIA-2 vs. MAIA (Not-Distracting: from 0.63 to 0.72; Not-Worrying: from 0.59 to 0.68), even though the sample recruited in our study was more severely depressed. However, reliability of the Not-Distracting and Not-Worrying scales were still at risk to undercut threshold values. Psychometric replication studies recruiting depressed samples are needed to clarify whether these scales require further modifications; for example, by avoiding use of reverse scored items that have been shown to negatively affect internal consistency estimates [66,67]–an effect which could be pronounced in severely affected patients due to cognitive impairments such as concentration difficulties. In conclusion, our findings suggest the use of MAIA-2 in future studies to ensure appropriate reliability particularly in clinically depressed samples, but future research should be aware of potential subthreshold internal consistencies regarding the Noticing, Not-Distracting, and Not-Worrying scales.

### 4.2 Correlational findings

Prior to treatment, inter-scale correlations were largely comparable with the English MAIA-2 validation study [35] except for Noticing and Not-Worrying showing a moderately negative correlation at admission, but not at discharge (Mehling et al. [35]: *r* = -.09). To the best of our knowledge, there is only one previous study recruiting depressed patients and reporting scale-scale correlations which found no significant correlation between both scales [68]. However, Forkmann et al. used the original MAIA, which may limit the comparability of the results. Our findings may be of clinical importance because the Not-Worrying scale assesses mental responses of worry or distress to sensations of pain or discomfort and is therefore conceptually related to pain catastrophizing [33,65]–a multifaceted construct defined as repetitive negative thinking in response to pain sensations by exhibiting feelings of helplessness, worry, and cognitive shifts to magnification of pain [69]. Thus, the tendency to catastrophize in severely depressed patients may be reflected by the correlation between hyper-awareness of body sensations and the susceptibility to worry about pain or unpleasant sensations. A possible link between interoception and intensified self-focus in depression may be of great clinical interest [16] as shifts to maladaptive self-referential cognitive styles predict prolonged episode duration [70] and play a major role in the onset of future depression [71]. It has also been shown that worry about physical sensations may be associated with increased suicidal ideation [68,72]. Conversely, post-treatment scores revealed a moderate positive correlation between Not-Worrying and Trusting, which was not found at admission, but may be indicative of an interrelatedness of both constructs in the post-treatment period. Both dimensions have recently been identified as independent predictors of residual symptoms of depression at the end of hospital treatment [14]. Regarding the post-treatment condition, the correlation matrix deviated from findings of Mehling et al.’s validation sample towards more positive bivariate associations, which might suggest different conceptualizations of the instrument’s dimensionality depending on the progress of treatment. Indeed, some authors have concluded from large inter-scale correlations that the number of questionnaire dimensions should be substantially reduced (e.g., see [34,73]). These questions should be addressed in a future formal assessment of MAIA score dimensionality in an MDD sample.

### 4.3 Responsiveness of MAIA-2

We investigated the responsiveness of MAIA-2 during hospital treatment. This represents an important contribution to the literature, which could inform future clinical research investigating changes in self-reported interoception in MDD samples and support future sample size estimations. As expected, significant improvements were identified on all scales with medium-to-large changes in regulatory or self-evaluative aspects of self-reported interoception, such as improving the capacity to cope with mental distress by focusing on body sensations, active listening to the body for insight, and the appraisal of somatic stimuli as trustworthy sources of information. These findings are congruent with previous research in the point that the largest improvements occurred on those dimensions which have been shown to be most affected in MDD [26,31]. However, the present study is limited by the fact that we cannot make clinical judgements on the interoceptive status of our sample because we did not recruit healthy matched controls. Nevertheless, post-treatment scores were largely comparable to previously published studies recruiting the general population [35,63] suggesting a normalization of impaired self-reported interoception during hospital treatment.

### 4.4 MIDs of MAIA-2 and depression-related treatment outcomes

Finally, we determined the minimal magnitude of change in multidimensional self-reported interoception that differentiated between treatment response groups by referring to anchor-based methods. In addition, we also explored MIDs using a distribution-based method. There were inconsistencies between the results of the different methods which are well known in the psychometric literature and which might be traced back to methodological characteristics of each approach (e.g., see [74]): For example, the 0.5*SD method has been related to the ability to discriminate between minimal perceivable differences instead of reflecting outcome-related differences [58]. In contrast, anchor-based approaches have the advantage of specifically answering questions about the minimal change that is associated with a clinical outcome. Whereas ROC derived MIDs may be applied to both the group and individual level, MIDs determined by the mean change method are only interpretable on the group level and are therefore not equivalent [74]. MIDs derived by the mean change method are usually based on small sample sizes leading to deflated power and unreliable estimates [74] as shown in our study by wide confidence intervals. Thus, MIDs were not aggregated for each scale (e.g., by the median) since they provide miscellaneous information. In this section, we will focus on the ROC curve derived cut-points which are more useful for clinical contexts (i.e., interpretable on the individual level) [74] by informing clinicians about changes in interoceptive facets that are associated with the occurrence of residual depressive symptoms–a unfavorable, but common condition in the treatment of MDD with adverse long-term outcomes [12]. Both correlational findings and AUC indicated that changes on the Noticing and Not-Distracting scales may not be considered as reasonable diagnostic classifiers for antidepressive responses and are therefore excluded from the discussion. For the binary outcome remission/no remission, the ROC findings demonstrated appropriate sensitivity (≥ .80) for three scales (Attention Regulation, Emotional Awareness, Self-Regulation) suggesting that patients who achieved remission could be accurately identified by the MID cut-points (≥ .71, ≥ .00, ≥ 1.00, respectively) reflecting improvements on the Likert scale (positive predictive values: 57.14%, 46.43%, 51.06%, respectively). Conversely, the results showed appropriate specificity (≥ .80) for two scales (Body Listening, Trusting) implying that corresponding MID cut-points better identified patients who did *not* achieve remission. This means that patients who exhibited subthreshold improvements on those dimensions (< 1.33 points) were at high risk for residual symptoms (negative predictive value for the two scales: 76.67%). However, small improvements on the Body Listening and Trusting scales (≥ .33 points) showed reasonable accuracy to correctly identify partial responders (positive predictive values: 82.81%, and 82.76%, respectively)–a clinical state that has been associated with improved quality of life, well-being, and functional status [43]. To sum up, the analyses identified a pattern of interoceptive changes that was associated with beneficial treatment outcomes. The main findings of the ROC curve analysis revealed the importance of improvements in regulative aspects of self-reported interoception, of a strengthened awareness of mind-body connection, and of increasing body confidence as key factors for achieving remission. There is also preliminary evidence that the detrimental sequelae of abnormalities in multidimensional self-reported interoception are independent of somatic symptom severity (e.g. pain) which also negatively influences treatment outcomes [14]. Our findings contribute to an abundance of outcome predictors that have been identified including clinical variables (e.g., episode duration, psychiatric comorbidity), psychosocial factors (e.g., age at onset, childhood maltreatment), neuroimaging findings (e.g., low volume of the hippocampus at baseline), inflammatory markers (e.g., CRP, IL-6, TNF-*α*), and (epi)genetic factors [75].

### 4.5 Clinical implications

Approximately one third of patients included in our naturalistic study were in remission and 39.08% partially responded to treatment, which is consistent with treatment outcomes from the STAR*D study [76]. Our findings may be of high clinical relevance for the development of new treatments that target the interoceptive system for managing residual symptoms of depression (which have been associated with detrimental clinical outcomes, reduced quality of life, and impaired daily functioning [13,14]). Available state-of-the-art pharmacological and psychotherapeutic treatments for MDD have several shortcomings in terms of efficacy, effectiveness, safety, tolerability, long-term outcome, or treatment specificity, and their evidence is limited by a small number of high-quality studies, by the susceptibility to publication bias (which might lead to an overestimation of treatment effects), and by low replicability of study findings [77–89]. Due to the high global prevalence of MDD [11], there is an urgent need for effective and safe therapies for MDD that specifically support the management of residual symptoms and increase remission rates. Therefore, the development of therapies targeting the impaired interoceptive system is currently a promising approach in psychiatry [3,90–92]. There is preliminary evidence that treatments with an interoceptive mechanism of action improve clinical states (e.g. chronic pain, anxiety, eating, substance use, and affective disorders [93]). So far, interoceptive treatments for MDD mainly focus on mindfulness-based interventions (e.g. [94,95]). This may be due to reports that mindfulness-based cognitive therapy prevents depression relapse in patients suffering from recurrent MDD [96] and effectively mitigates residual symptoms of depression [97] probably through an interoceptive mechanism of action [32,33]. However, mindfulness research has repeatedly been criticized for poor methodological quality [98,99], for overestimating clinical effects [100], and for underreporting adverse reactions [101]. Given the current “mindfulness hype” [102] in translational interoception research, we must acknowledge the limited evidence, safety, and suitability of mindfulness-based interventions for patients suffering from severe MDD. These patients regularly exhibit concentration difficulties, brooding rumination, or a history of suicidality, all of which could impede or contraindicate periodic mindfulness practice [103–105]. Indeed, given the high prevalence of mindfulness training-related adverse reactions [105,106] the clinical use of established mindfulness-based treatments is opposed in severely depressed patients because an aggravation of the disorder cannot be excluded [105]. Reported adverse reactions of mindfulness-based interventions include mania [107], psychosis [108], suicidal ideation [105], depersonalization/derealization [109], anxiety [110], panic reactions, negative feelings, or sleep disturbances [111]. Furthermore, the Eastern basis of mindfulness-based methods [111,112] may conflict with the spiritual backgrounds and needs of Western patients, which is a further limitation for the broad application of these techniques in clinical psychiatry.

Apart from that, depressed women frequently use touch-based complementary treatments (e.g., massage therapy), particularly when reporting a poor self-rated health status [113]. The female preference for body (psycho)therapy may be understood in the light of abnormal interoceptive signaling, whereby women regularly report higher maladaptive attention to interoceptive states, more somatic symptoms, and lower interoceptive accuracy compared to men [114]. There is increasing evidence that treatments applying affective touch (i.e. gentle, caress-like stroking touch [115]) have antidepressive, anxiolytic, analgesic, and stress-relieving effects [116–122]. A meta-analysis showed the highest effectiveness of massage therapy with moderate to large effect sizes when two treatments are given per week over a five-week period with a minimum duration per session of 30 minutes [116]. Different mechanisms of action have been proposed which may explain the antidepressive effects of affective touch–one of them refers to the interoceptive nervous system [123]: The mammalian non-glabrous skin contains unmyelinated low-threshold C tactile (CT) mechanoreceptors that optimally respond to light touch in a velocity range of 1–10 cm/s [115]. The activation of CT afferents through affective touch is accompanied by a positive affective state of well-being that has been associated with an activation of the insular cortex and other interoception-related neural structures [124]. CT-mediated touch may therefore be conceptualized as an interoceptive antagonist of MDD-related anhedonia [123]. Preliminary evidence points to the human skin as a safe gateway to externally modulate interoceptive states, e.g. by increasing heartbeat perception accuracy [125] or a sense of body ownership [126]. However, there is a significant lack of research translating findings about the interoceptive basis of affective touch into clinical psychiatric practice [127].

### 4.6 Limitations

The present study is subject to several limitations. First, we focused on a clinimetric validation [128] of the MAIA-2 rather than establishing the measure’s construct validity and test-retest reliability. Studies are needed that investigate further psychometric properties of the updated German version of MAIA-2. Second, we used a pre-post design which limits decisive conclusions about causal relationships regarding potential impacts of abnormal multidimensional self-reported interoception on MDD. Replications are therefore needed using several measurement points to clarify mechanisms of action in randomized controlled trials. Third, a large proportion of participants were severely depressed and exhibited relatively low scores on some MAIA-2 dimensions at study inclusion, which might result in a ‘regression to the mean’ effect (extreme values tend to increase/decrease on further measurement occasions) limiting reliability of the sensitivity to change analysis. Fourth, we determined anchor-based MIDs by defining ‘clinical importance’ as favorable treatment outcome instead of using a global transition item that better reflects patient-reported evaluations of minimal change and may therefore produce different cut-points [59]. Due to the small sample size, we were not able to conduct separate analyses for women and men, although effects of sex on interoception are discussed in the literature [14,114]. Finally, the pre-post analysis could be biased by response shifts which have never been investigated in interoceptive self-report measures but frequently occur on interoception-related constructs such as fatigue, pain, or well-being across changing health conditions [129].

### 4.7 Conclusions

The present study demonstrated the applicability of the MAIA-2 questionnaire in a severely depressed sample. The updated version may have led to reliability improvements regarding the revised scales and therefore future use of the original MAIA should be avoided. However, clinicians using MAIA-2 in severely depressed samples should pay special attention to potential subthreshold internal consistencies on three scales (Noticing, Not-Distracting, Not-Worrying). The measure’s dimensions were sensitive to change and MIDs could be established that corresponded with antidepressive treatment outcomes. Our findings are consistent with a growing area of research which considers somatic feelings as key contributors to mental health.

## Supporting information

S1 Questionnaire German version of the MAIA-2 questionnaire. (PDF)
