# Supplementary material for: Validation of the Multidimensional Assessment of Interoceptive Awareness (MAIA-2) questionnaire in hospitalized patients with major depressive disorder
Source: PLoS One. 2021 Jun 25;16(6):e0253913. doi: 10.1371/journal.pone.0253913 (PMC8232409; doi:10.1371/journal.pone.0253913)
Supplement: S1 Questionnaire — (PDF) [file pone.0253913.s001.pdf]

Multidimensional Assessment of Interoceptive Awareness, Version 2 (MAIA-2)

Kontakt:
Wolf E. Mehling, MD
Osher Center for Integrative Medicine
University of California, San Francisco
1545 Divisadero St., 4th floor
San Francisco, CA 94115
Phone: 01 (415) 353 9506
mehlingw@ocim.ucsf.edu
http://www.osher.ucsf.edu/maia/

Übersetzung von MAIA-2 in Kooperation mit Wolf E. Mehling (basierend auf der dt. Erstversion von Boris Bornemann, Max-Planck-Institut, Leipzig, Dezember 2012):
Michael Eggart
Hochschule Ravensburg-Weingarten
University of Applied Sciences
Fakultät Soziale Arbeit, Gesundheit und Pflege
Leibnizstr. 15
D-88250 Weingarten
Telefon: +49 751 501 9467
michael.eggart@rwu.de
https://www.researchgate.net/profile/Michael_Eggart

Multidimensional Assessment of Interoceptive Awareness (Version 2)

Nutzungsrecht und Copyright

Der MAIA-2 ist urheberrechtlich geschützt. Gleichwohl ist seine Nutzung gebührenfrei und bedarf keiner ausdrücklichen schriftlichen Genehmigung. Mit seiner Nutzung erkennt man die folgenden Regeln an:

- Bei Bezugnahme auf den Fragebogen bitten wir den vollständigen Namen zu nennen (Multidimensional Assessment of Interoceptive Awareness, Version 2) und eine entsprechende Zitation vorzunehmen.
- Modifikationen sind ohne unser schriftliches Einverständnis erlaubt. Allerdings bitten wir darum, dass jegliche Veränderung in den entsprechenden Publikationen klargestellt und uns mitgeteilt wird.
- Falls Sie Teile des MAIA-2 benutzen wollen, empfehlen wir, nicht Einzelfragen, sondern komplette Subskalen zu verwenden, damit die psychometrischen Eigenschaften erhalten bleiben.
- Wenn Sie den MAIA-2 in eine andere Sprache übersetzen, dann senden Sie uns bitte eine Kopie.
- Falls sich andere Kollegen für den MAIA-2 interessieren, verweisen Sie bitte auf die Originalpublikation (Mehling WE et al. (2018) PLoS ONE 13(12): e0208034. doi:10.1371/journal.pone.0208034) und die Website www.osher.ucsf.edu/maia/, damit sie die jüngste Version und Scoring-Anleitung erhalten.

Scoring-Anleitung

Berechnen Sie das arithmetische Mittel der Items separat für jede Subskala. Die Werte für die Items 5, 6, 7, 8, 9 und 10 der Subskala Nicht-Ablenken sowie die Werte für die Items 11, 12 und 15 der Subskala Sich-Keine-Sorgen-Machen werden umgepolt (U). Formel: 5 - Item-Score.

1. **Bemerken:** Gewahrsein von unangenehmen, angenehmen und neutralen Körperempfindungen
   (I1_____+ I2_____+ I3_____+ I4_____) / 4 = _____

2. **Nicht-Ablenken:** Tendenz, Schmerzen oder unangenehme Empfindungen nicht zu ignorieren oder sich nicht abzulenken
   (I5(U) _____+ I6(U) _____+ I7(U) _____+ I8(U) _____+ I9(U) _____+ I10(U) _____) / 6 = _____

3. **Sich-Keine-Sorgen-Machen:** Tendenz, sich bei Schmerzen oder unangenehmen Empfindungen keine Sorgen zu machen und nicht in emotionalen Stress zu geraten
   (I11(U) _____+ I12(U) _____+ I13_____+ I14_____+ I15(U) _____) / 5 = _____

4. **Aufmerksamkeitsregulation:** Fähigkeit, die Aufmerksamkeit auf Körperempfindungen zu richten und aufrechtzuerhalten
   (I16_____+ I17_____+ I18_____+ I19_____+ I20_____+ I21_____+ I22_____) / 7 = _____

5. **Emotionales Gewahrsein:** Gewahrsein eines Zusammenhangs zwischen Körperempfindungen und Emotionen
   (I23_____+ I24_____+ I25_____+ I26_____+ I27_____) / 5 = _____

6. **Selbstregulation:** Fähigkeit, Disstress zu regulieren, indem die Aufmerksamkeit auf Körper-empfindungen gerichtet wird
   (I28_____+ I29_____+ I30_____+ I31_____) / 4 = _____

7. **Auf-den-Leib-Hören:** Aktives Hören auf den Körper, um von ihm zu lernen
   (I32_____+ I33_____+ I34_____) / 3 = _____

8. **Vertrauen:** Den eigenen Leib als sicher und vertrauenerweckend erfahren
   (I35_____+ I36_____+ I37_____) / 3 = _____

Unten finden Sie eine Liste von Aussagen. Geben Sie bitte für jede der folgenden Aussagen an, wie oft sie generell im täglichen Leben auf Sie zutrifft.

	Kreisen Sie in jeder Reihe eine Zahl ein.					
	**Nie**					**Immer**
1. Wenn ich angespannt bin, merke ich, wo in meinem Körper die Anspannung auftritt.	0	1	2	3	4	5
2. Ich merke es, wenn ich mich in meinem Körper nicht wohlfühle.	0	1	2	3	4	5
3. Ich merke, wo in meinem Körper ich mich wohlfühle.	0	1	2	3	4	5
4. Ich bemerke Veränderungen in meiner Atmung, zum Beispiel, ob ich langsamer oder schneller atme.	0	1	2	3	4	5
5. Ich ignoriere körperliche Anspannung oder Unwohlsein, bis diese stärker werden.	0	1	2	3	4	5
6. Ich lenke mich von unangenehmen Empfindungen ab.	0	1	2	3	4	5
7. Wenn ich Schmerz oder Unbehagen empfinde, versuche ich mich durchzubeißen.	0	1	2	3	4	5
8. Ich versuche, Schmerzen zu ignorieren.	0	1	2	3	4	5
9. Ich verdränge unangenehme Gefühle, indem ich mich auf etwas anderes konzentriere.	0	1	2	3	4	5
10. Wenn ich unangenehme Körperempfindungen spüre, dann beschäftige ich mich mit etwas anderem, um sie nicht spüren zu müssen.	0	1	2	3	4	5
11. Wenn ich körperliche Schmerzen habe, ärgere ich mich.	0	1	2	3	4	5
12. Wenn ich mich unwohl fühle, fange ich an mir Sorgen zu machen, dass irgendetwas nicht stimmt.	0	1	2	3	4	5
13. Ich kann unangenehme Körperempfindungen spüren, ohne dass sie mich beunruhigen.	0	1	2	3	4	5
14. Ich kann ruhig und unbesorgt bleiben, wenn ich mich unwohl fühle oder Schmerzen habe.	0	1	2	3	4	5
15. Wenn ich Unbehagen oder Schmerzen empfinde, geht es mir nicht aus dem Kopf.	0	1	2	3	4	5
16. Ich kann auf meine Atmung achten ohne von dem, was um mich herum geschieht, abgelenkt zu werden.	0	1	2	3	4	5
17. Ich kann meiner inneren Körperempfindungen gewahr bleiben, auch wenn um mich herum eine Menge los ist.	0	1	2	3	4	5
18. Ich kann auf meine Körperhaltung achten, während ich mich mit jemandem unterhalte.	0	1	2	3	4	5

Kreisen Sie in jeder Reihe eine Zahl ein.	Nie					Immer
19. Wenn ich abgelenkt bin, kann ich mit meiner Aufmerksamkeit zu meinem Körper zurückkehren.	0	1	2	3	4	5
20. Ich kann meine Aufmerksamkeit vom Denken auf das Spüren meines Körpers zurücklenken.	0	1	2	3	4	5
21. Ich kann den gesamten Körper auch dann weiter bewusst wahrnehmen, wenn ich in einem Teil Schmerz oder Unbehagen empfinde.	0	1	2	3	4	5
22. Ich kann meine Aufmerksamkeit bewusst auf meinen Körper als Ganzes richten.	0	1	2	3	4	5
23. Ich bemerke, wie mein Körper sich verändert, wenn ich wütend bin.	0	1	2	3	4	5
24. Wenn etwas in meinem Leben nicht stimmt, kann ich das in meinem Körper spüren.	0	1	2	3	4	5
25. Ich merke, dass mein Körper sich anders anfühlt, wenn ich etwas Friedliches und Entspannendes erlebe.	0	1	2	3	4	5
26. Ich merke, dass meine Atmung freier und leichter wird, wenn ich mich wohlfühle.	0	1	2	3	4	5
27. Ich merke, wie mein Körper sich verändert, wenn ich glücklich oder fröhlich bin.	0	1	2	3	4	5
28. Wenn mir alles zu viel wird, kann ich einen Ort der Ruhe in mir finden.	0	1	2	3	4	5
29. Wenn ich meine Aufmerksamkeit auf meinen Körper richte, empfinde ich ein Gefühl innerer Ruhe.	0	1	2	3	4	5
30. Ich kann meinen Atem dazu benutzen, innere Spannungen abzubauen.	0	1	2	3	4	5
31. Wenn ich in meine Gedanken verstrickt bin, kann ich meinen Geist beruhigen, indem ich auf Körper und Atem achte.	0	1	2	3	4	5
32. Ich höre auf meinen Körper, was er über meine emotionale Verfassung sagt.	0	1	2	3	4	5
33. Wenn ich aufgebracht bin, nehme ich mir Zeit herauszufinden, wie mein Körper sich anfühlt.	0	1	2	3	4	5
34. Ich höre auf meinen Körper, um zu erkennen, was zu tun ist.	0	1	2	3	4	5
35. Ich bin in meinem Körper zu Hause.	0	1	2	3	4	5
36. Ich empfinde meinen Körper als einen sicheren Ort.	0	1	2	3	4	5
37. Ich vertraue meinen Körperempfindungen.	0	1	2	3	4	5
